# Economic costs and medications for diabetes in older patients in Beijing, China: electronic insurance data analysis

**DOI:** 10.3389/fphar.2025.1549244

**Published:** 2025-04-07

**Authors:** Yanhua Ma, Yan Zhao, Ran Wei, Jingtao Qiao, Jingyi Luo, Lina Zhang, Jie Zhang, Mingqun Deng, Yang Yu, Weihao Wang, Qi Pan, Lixin Guo

**Affiliations:** ^1^ Department of Endocrinology, Beijing Hospital, National Center of Gerontology, Institute of Geriatric Medicine, Chinese Academy of Medical Sciences, Beijing, China; ^2^ Graduate School of Peking Union Medical College, Chinese Academy of Medical Sciences, Beijing, China

**Keywords:** diabetes, cost, medication, electronic insurance database, older patients

## Abstract

**Background:**

With the aging of population, the proportion of elderly patients with diabetes is gradually increasing, which poses challenges in the management and treatment of diabetes in this population.

**Methods:**

The aim of the study was to investigate the temporal changes in the treatment regimens and medical expenditures in older patients with diabetes in Beijing, China. Data of patients with diabetes from the Beijing Medical Insurance Database with medical records from 2016 to 2018 were retrospectively analyzed. Primary and secondary outcomes included the number of medications, comorbidities, diabetes-related complications, the estimated annual drug cost, the treatment strategies for elderly diabetic patients, and the classes of drugs prescribed.

**Results:**

Data of 598,440 patients with diabetes in 2018 revealed that 49.8% of the recruited patients were female among elderly patients (>65 years old). The most common comorbidity was hypertension (87.6%). Over the 3 years, about 4.51 medications, including 1.88 antiglycemic drugs and 2.63 non-antiglycemic drugs were prescribed in elderly patients. The mean total annual medication cost was ¥12,186 ($1,676), including ¥6,116 ($841) for antiglycemic drugs and ¥6,070 ($835) for non-antiglycemic drugs. Hypertension (cost ¥4,658, $640, mean medications 2.12 for elderly patients), dyslipidemia (¥5,044, $693, 1.70), and coronary heart disease (¥4,004, $550, 1.40) were the top three diseases that caused the increase in the cost and medications. Over the 3 years, more than 94% of elderly diabetic patients received at least one type of antiglycemic drugs, and the α-glucosidase inhibitors and premixed insulin are the most commonly prescribed hypoglycemic drugs and insulin, respectively.

**Conclusion:**

Diabetes management in older patients faces challenges due to extensive variability. Medication analysis in this study found that the current situation of comprehensive control of diabetes in elderly patients is worrying, and the complexity of their medication is still on the increasing trend. It is important to select more appropriate antiglycemic drugs to economically benefit the patients and to control the progression of complications.

## 1 Introduction

Diabetes has been one of the four dominating non-communicable diseases worldwide. According to the data in 2019, the number of elderly diabetic patients (≥65 years old) in China is about 35.5 million, ranking first globally and accounting for 1/4 of global elderly diabetic patients (Sinclair et al., 2020). With the aging of population, the current treatment and management of elderly diabetic patients are critical.

The global costs of diabetes and its complications are predicted to be increasing by 2030. Increased diabetes costs will share the global gross domestic product from 1.8% in 2015 to 2.2% in 2030 (Bommer et al., 2018). Notably, indirect costs of diabetes account for about 34.7% of total costs. However, the economic cost percentage in the middle-income was more prominent than in high-income countries (Bommer et al., 2017). Medical insurance costs for patients with diabetes are two to three times more than for those without (Li et al., 2019). Type 2 diabetes-related cost made up about 40.6% of the overall cost in rural China (Wu et al., 2019). In 2008, diabetes-related expenditure accounted for about 13% in China (Li et al., 2019). Then a new medical insurance reform was started in 2009. As we previously described, there was a noticeable reduction in the costs and medications for diabetes, including antiglycemic and non-antiglycemic drugs. The estimated decreased annual cost of drugs was also observed (Guo et al., 2021). Yang’s study revealed that the estimated annual cost in elderly diabetic patients was more than that in younger patients (Yang et al., 2012).

Antiglycemic regimens for patients with type 2 diabetes have changed significantly in recent years. In 2018, about 84.7% of diabetic patients received at least one antiglycemic drug in Beijing, China (Guo et al., 2021). Unlike the guideline suggesting that metformin should be considered as the basic treatment, α-glucosidase inhibitors were the most commonly used hypoglycemic drug for Chinese people with diabetes (Guo et al., 2021). The health status of elderly diabetic patients varies significantly among individuals, often accompanied by varying degrees of cognitive dysfunction and complex underlying diseases. The principles of drug treatment for elderly diabetic patients include (Guo and Xiao, 2024): (1) Give priority to drugs with lower risk of hypoglycemia; (2) Choose simple and highly compliant drugs to reduce the risk of multiple drugs; (3) Weigh the benefit-risk ratio and avoid excessive treatment; and (4) Pay attention to factors such as liver and kidney functions, heart function, complications, and accompanying diseases. Previous studies have revealed that the compliance rate diabetic patients is still low despite polypharmacy (Ruan et al., 2016; Lipska et al., 2016). However, there is still no large-scale study to explore the cost and medications in elderly diabetic patients.

This study analyzed 3 years’ trends in medication cost and treatment costs for the elderly diabetic patients in Beijing’s Medical Insurance Database.

## 2 Materials and methods

### 2.1 Study design and ethical approval

A retrospective, observational, and multi-center study was carried out to estimate the temporal changes in the drug regimens and cost in elderly patients with diabetes in China over 3 years from 2016 to 2018. The study was approved by the Ethics Committee of Beijing Hospital (No.2021BJYYEC-022-01).

### 2.2 Study population and data collection

Patients with diabetes in Beijing’s Medical Insurance Database with medication records from 2016 to 2018 were included in this study. The inclusion criteria were as our previous study (Guo et al., 2021). According to Beijing’s medical insurance policy, medication can be prescribed for each patient in less than a month, so if patients take medication regularly, it needs to be prescribed within 2 months. The exclusion criteria for this study were as follows: (1) Did not have a diagnosis of diabetes, and (2) Did not have a sequential prescription record (2 months interval). The cohorts included 897,385 patients in 2016; 959,509 patients in 2017; and 996,142 in 2018. Of these, 598,440 patients consecutively appeared over 3 years.

Complications and Comorbidities were defined using ICD codes. Demographic data, including age, sex, medications, costs, prescriptions, comorbidities and diabetes-related complications were extracted from the Beijing Medical Insurance Database. Comorbidities in people with diabetes include hypertension, coronary heart disease, dyslipidemia, stroke, chronic lung disease, and osteoporosis. Diabetes-related complications include diabetic peripheral neuropathy, diabetic retinopathy, and diabetic nephropathy. We collected information from the Beijing Municipal Medical Insurance Database, and appropriately encrypted any information related to patient personal information or privacy in the data to ensure that it does not contain any other sensitive information that can directly or indirectly expose the patient’s identity. Each prescription has a unique serial number in the database.

### 2.3 Primary outcomes and definitions used

Primary outcomes in this study embraced the cost and prescriptions of diabetic patients from 2016 to 2018. Interested endpoints included the number of medications and comorbidities, the estimated annual drug regimen cost, the treatment strategies for elderly diabetic patients, and the classes of drugs prescribed. Any prescriptions for short-term use like antibiotics were excluded from this study.

The description of antiglycemic and non-antiglycemic drug medications was conducted by drug class for each study year. Specific classes and the definitions of monotherapy, and combination therapy were as illustrated previously (Guo et al., 2021): Drug classes included in medical insurance were metformin, α-glucosidase inhibitors, dipeptidyl peptidase-4 inhibitors, insulin, sulfonylureas, prandial glucose regulators (glinides) and thiazolidinediones. A patient was considered receiving any antiglycemic drug if they had been treated with at least 1 antiglycemic drug prescription in each study year. Monotherapy means patients received only 1 recorded antiglycemic drug prescription in the past 1 year. Oral combination therapy means patients received 2 or more oral antiglycemic drug prescriptions in different classes in the past 1 year. Oral drug and insulin combination therapy indicates that patients received at least 1 oral antiglycemic drug prescription and at least 1 insulin prescription in the past 1-year period. The summary of drug medications from included patients was performed by two statisticians and two clinicians.

### 2.4 Statistical analysis

The distributions of the diagnoses, the number of medications and the estimated annual cost were calculated by the Wilcoxon rank sum test. Fisher’s exact test and χ2 were used as tests of significance for categorical variables. The estimated annual cost was log-transformed. A smearing estimator was chosen to correct for retransformation bias for the heteroscedasticity. To adjust the estimated annual cost and the number of medications by demographic, diabetic complication and medical history, multivariable regression models included covariables such as sex, age, comorbidities, and a variable for the year of sampling to evaluate the changes over time. Statistical analysis was performed using the SAS software, version 9.4 (SAS Institute, Inc.), P < 0.05 was considered to indicate statistical significance. All the statistical methods were described in our previously published article (Guo et al., 2021).

## 3 Results

### 3.1 Demographics of the included population

Data of 598,440 patients in 2018 revealed that 49.8% of the recruited patients were female among the elderly patients (>65 years old). The most common comorbidities analyzed in this project were hypertension (87.6%), coronary heart disease (82%), dyslipidemia (74.1%), stroke (49.1%), chronic lung disease (43.3%), and osteoporosis (33%) in the elderly. The most common complications were diabetic peripheral neuropathy (31.5%), diabetic retinopathy (12%), and diabetic nephropathy (11.3%) in the elderly (Supplementary Table S1).

### 3.2 Changes in medications, disease and cost

Over the 3 years, about 4.51 medications, including 1.88 antiglycemic drugs and 2.63 non-antiglycemic drugs were prescribed in elderly patients. There was a mean of 4.35 comorbidities, including 0.65 glycemic diseases and 3.70 non-glycemic diseases in the elderly patients who were treated. The mean total annual medication cost was ¥12,186 ($1,676), including ¥6,116 ($841) for antiglycemic drugs and ¥6,070 ($835) for non-antiglycemic drugs in elderly patients. The mean annual cost per medication was ¥2,734 ($376) for elderly patients. However, the prices of antiglycemic drugs were higher than non-antiglycemic drugs. Over the 3 years, although the number of medications was increasing, the total annual drug costs in 2017 and 2018 decreased compared with that in 2016 in the elderly patients (Table 1).

**TABLE 1:** Changes in medications, comorbidities, and costs of diabetes patients in aged >65 years of age, 2016-2018.

	Mean for 2016-2018	Mean for each year			Change from 2016 to 2017 no. (%)	Change from 2016 to 2018 no. (%)
		2016	2017	2018		
Number of medications	4.51	4.41	4.57	4.56	0.16 (3.5)	0.15 (3.3)
Antiglycemic drugs	1.88	1.80	1.89	1.94	0.09 (5.1)	0.14 (7.8)
Non-antiglycemic drugs	2.63	2.61	2.67	2.62	0.06 (2.5)	0.01 (0.2)
Number of comorbidities	4.35	4.36	4.35	4.34	−0.01 (−0.3)	−0.03 (−0.7)
Glycemic diseases	0.65	0.65	0.65	0.64	0.00 (−0.6)	−0.01 (−1.3)
Non-glycemic diseases	3.70	3.71	3.70	3.69	−0.01 (−0.2)	−0.02 (−0.6)
Total annual drug cost, ¥	12,186	12,833	11,782	11,944	−1,051 (−8.2)	−889 (−6.9)
Antiglycemic drugs	6,116	6,277	5,933	6,138	−344 (−5.5)	−139 (−2.2)
Non-antiglycemic drugs	6,070	6,556	5,849	5,805	−707 (−10.8)	−751 (−11.4)
Total annual cost/drug, ¥	2,734	2,929	2,611	2,661	−318 (−10.8)	−268 (−9.1)
Cost/antiglycemic drug	3,061	3,247	2,954	2,984	−293 (−9.0)	−263 (−8.1)
Cost/non-antiglycemic drug	1977	2,131	1887	1914	−244 (−11.5)	−217 (−10.2)

### 3.3 Cost of medication associated with diseases and comorbidities

Hypertension (cost ¥4,658, $640, mean medications 2.12 for elderly patients), dyslipidemia (¥5,044, $693,1.70), and coronary heart disease (¥4,004, $550,1.40) were the top three diseases that caused the increase in the cost and medications. Diabetic patients without comorbidity consumed a mean annual cost of ¥7,951 ($1,093) and mean medications of 2.34 in the elderly patients. However, the greater the number of comorbidities in diabetic patients, the higher the number and cost of medications. When a maximum of 6 types of comorbidities were combined, the number of medications could be increased to 5.97 in the elderly patients, and the annual medication cost could be expanded to ¥17,504 ($2,407) in the elderly patients (Table 2).

**TABLE 2:** Number and cost of medications in stratified patient groups.

Patient group	Parameter	≥65				
		n	Mean	Adjusted mean	SD	Difference compared with reference
Sex						
Male	No. of medications	392,493	4.52	5.06	2.33	Ref
	Cost ¥	392,493	12,076	14,216	10,266	Ref
Female	No. of medications	392,025	4.52	5.10	2.32	0.04
	Cost ¥	392,025	12,243	14,380	10,212	164
Hypertension						
Absent	No. of medications	95,282	2.64	3.16	1.50	Ref
Present	No. of medications	689,236	4.78	5.28	2.30	2.12
Absent	Cost ¥	95,282	8,185	10,077	9,064	Ref
Present	Cost ¥	689,236	12,709	14,735	10,271	4,658
Coronary heart disease						
Absent	No. of medications	139,600	3.24	3.86	1.84	Ref
Present	No. of medications	644,918	4.79	5.26	2.33	1.40
Absent	Cost ¥	139,600	8,669	10,813	8,868	Ref
Present	Cost ¥	644,918	12,915	14,817	10,359	4,004
Dyslipidemia						
Absent	No. of medications	203,566	3.24	3.72	1.79	Ref
Present	No. of medications	580,952	4.97	5.42	2.32	1.70
Absent	Cost ¥	203,566	8,558	10,265	8,230	Ref
Present	Cost ¥	580,952	13,422	15,309	10,569	5,044
Stroke						
Absent	No. of medications	396,156	4.24	4.86	2.27	Ref
Present	No. of medications	388,362	4.80	5.24	2.35	0.38
Absent	Cost ¥	396,156	11,214	13,653	9,764	Ref
Present	Cost ¥	388,362	13,125	14,753	10,616	1,100
Chronic lung disease						
Absent	No. of medications	443,859	4.38	4.91	2.29	Ref
Present	No. of medications	340,659	4.70	5.26	2.36	0.35
Absent	Cost ¥	443,859	11,612	13,568	10,053	Ref
Present	Cost ¥	340,659	12,873	15,069	10,434	1,501
Osteoporosis						
Absent	No. of medications	524,302	4.41	4.98	2.31	Ref
Present	No. of medications	260,216	4.75	5.24	2.34	0.26
Absent	Cost ¥	524,302	11,718	13,895	9,969	Ref
Present	Cost ¥	260,216	13,049	14,953	10,708	1,058
Diabetic peripheral neuropathy						
Absent	No. of medications	534,920	4.35	4.97	2.27	Ref
Present	No. of medications	249,598	4.88	5.26	2.39	0.29
Absent	Cost ¥	534,920	11,530	13,873	9,823	Ref
Present	Cost ¥	249,598	13,509	15,012	10,958	1,139
Diabetic nephropathy						
Absent	No. of medications	695,106	4.45	4.88	2.30	Ref
Present	No. of medications	89,412	5.04	5.31	2.48	0.43
Absent	Cost ¥	695,106	11,795	13,200	9,901	Ref
Present	Cost ¥	89,412	14,993	15,923	12,198	2,722
Diabetic neuropathy						
Absent	No. of medications	690,667	4.43	4.82	2.29	Ref
Present	No. of medications	93,851	5.17	5.32	2.47	0.50
Absent	Cost ¥	690,667	11,846	13,448	10,025	Ref
Present	Cost ¥	93,851	14,469	15,095	11,435	1,647
No. of comorbidities						
0	No. of medications	14,075	1.89	2.34	1.01	Ref
	Cost ¥	14,075	6,586	7,951	10,058	Ref
1	No. of medications	36,884	2.58	2.93	1.43	0.59
	Cost ¥	36,884	7,242	8,601	8,521	650
2	No. of medications	83,067	3.28	3.69	1.75	1.35
	Cost ¥	83,067	8,609	10,415	8,172	2,464
3	No. of medications	183,726	4.29	4.65	2.18	2.32
	Cost ¥	183,726	11,068	12,831	9,248	4,880
4	No. of medications	239,229	4.83	5.21	2.26	2.87
	Cost ¥	239,229	12,862	14,546	10,172	6,596
5	No. of medications	171,991	5.22	5.59	2.32	3.25
	Cost ¥	171,991	14,339	15,777	10,783	7,826
6	No. of medications	55,546	5.58	5.97	2.41	3.63
	Cost ¥	55,546	15,986	17,504	11,842	9,554
No. of complications						
0	No. of medications	439,363	4.25	4.25	2.23	Ref
	Cost ¥	439,363	11,092	11,092	9,369	Ref
1	No. of medications	221,164	4.64	4.64	2.33	0.39
	Cost ¥	221,164	12,724	12,725	10,555	1,632
2	No. of medications	89,737	5.09	5.09	2.44	0.84
	Cost ¥	89,737	14,462	14,461	11,708	3,369
3	No. of medications	29,567	5.53	5.53	2.46	1.28
	Cost ¥	29,567	15,938	15,939	11,986	4,846
4	No. of medications	4,687	5.90	5.91	2.50	1.65
	Cost ¥	4,687	17,663	17,664	13,698	6,572

### 3.4 Changes in treatment regimens

Over the 3 years, more than 94% of elderly diabetic patients received at least one type of antiglycemic drug. Whether in monotherapy or combination therapy, the α-glucosidase inhibitors were the most commonly used regimen followed by metformin, sulfonylureas, premixed insulin glinides and dipeptidyl peptidase 4 inhibitors (DPP-4i) alone in the elderly (Table 3).

**TABLE 3:** Three-year changes in diabetes treatment regimens.

Variable	Medications	≥65		
		2016 (%)	2017 (%)	2018 (%)
Receiving any diabetes drug	No	14,392 (6.0)	13,151 (5.0)	14,241 (5.0)
	Yes	223,648 (94.0)	247,949 (95.0)	271,137 (95.0)
Monotherapy	Yes	82,323 (34.6)	84,400 (32.3)	87,847 (30.8)
α-glucosidase inhibitors	Yes	33,853 (14.2)	34,397 (13.2)	34,823 (12.2)
Metformin	Yes	18,606 (7.8)	20,138 (7.7)	22,341 (7.8)
Sulfonylureas	Yes	8,973 (3.8)	8,685 (3.3)	8,152 (2.9)
Premixed insulin	Yes	12,378 (5.2)	12,310 (4.7)	12,506 (4.4)
DPP-4i	Yes	0 (0.0)	400 (0.2)	1,201 (0.4)
Glinides	Yes	3,272 (1.4)	3,024 (1.2)	2,735 (1.0)
Oral combination therapy	Yes	103,861 (43.6)	122,276 (46.8)	139,027 (48.7)
α-glucosidase + Metformin	Yes	18,943 (8.0)	22,854 (8.8)	25,770 (9.0)
α-glucosidase + sulfonylureas	Yes	19,876 (8.3)	19,870 (7.6)	19,245 (6.7)
Metformin + sulfonylureas	Yes	11,448 (4.8)	12,503 (4.8)	12,988 (4.6)
α-glucosidase + Metformin + sulfonylureas	Yes	14,270 (6.0)	16,862 (6.5)	18,180 (6.4)
Metformin + DPP-4i	Yes	0 (0.0)	609 (0.2)	1883 (0.7)
Metformin + Glinides	Yes	3,420 (1.4)	3,476 (1.3)	3,470 (1.2)
α-glucosidase + Glinides	Yes	4,199 (1.8)	4,112 (1.6)	3,839 (1.3)
Oral + Insulin	Yes	22,176 (9.3)	28,473 (10.9)	34,118 (12.0)
α-glucosidase + Premixed insulin	Yes	11,497 (4.8)	12,338 (4.7)	12,447 (4.4)
α-glucosidase + Metformin + insulin	Yes	5,692 (2.4)	7,337 (2.8)	8,162 (2.9)
Metformin + Premixed insulin	Yes	5,054 (2.1)	5,700 (2.2)	6,143 (2.2)

DPP-4i: Dipeptidyl peptidase 4 inhibitor.

Premixed insulin was still the most frequently used insulin for monotherapy in diabetic patients. For monotherapy, metformin and DPP-4i were the only two types of drugs that were not decreasing for treatment in the elderly. Over the 3 years, the number of patients for monotherapy was gradually decreasing. However, the number of patients with oral combination therapy and oral drugs combined with insulin therapy was growing, which meant that the number and mode of medication for diabetes patients in China had increased.

### 3.5 Trends in the prescriptions of antiglycemic and non-antiglycemic drugs

**FIGURE 1:**
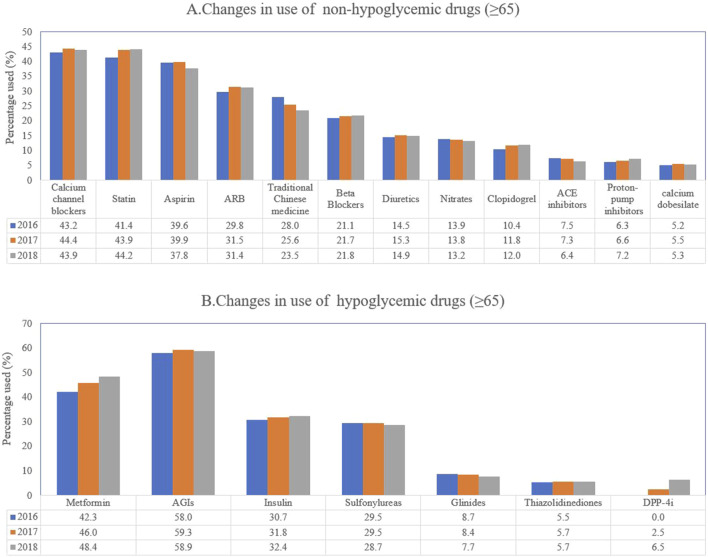
Trends of the prescriptions of antiglycemic and non-antiglycemic drugs. **(A)** changes in use of non-hypoglycemic drugs (≥65 years of age); **(B)** changes in use of hypoglycemic drugs (≥65 years of age).

Among the antiglycemic drugs, the α-glucosidase inhibitors were still the most prescribed drug, accounting for 58.9% in 2018, followed by metformin (48.4%), insulin (32.4%), sulfonylureas (28.7%), glinides (7.7%), thiazolidinediones (5.7%) and DPP-4i (6.5%) in the elderly. However, results revealed that the prescriptions including metformin (from 42.3% in 2016 to 48.4% in 2018), DPP-4i (from 0% to 6.5%), or insulin (from 30.7% to 32.4%) were gradually increasing over the 3 years in the elderly (Figure 1).

For the insulin regimen, premixed insulin (56.2% for elderly patients) was still the most prescribed insulin, followed by long-acting insulin (26.5%), and intermediate-acting insulin (13.2%) in 2018. However, fast-acting insulin was prescribed less than other types of insulin in the elderly. Over the 3 years, the regimens of fast-acting insulin (from 7.7% to 11.3% in the elderly) and long-acting insulin (from 20.2% to 26.5%) rose significantly. However, the regimens of short-acting insulin, intermediate-acting insulin and premixed insulin decreased significantly (Supplementary Table S2).

For non-antiglycemic drugs, statin (44.2%) was the most prescribed drug in 2018 followed by calcium channel blockers (43.9%), aspirin (37.8%), angiotensin receptor blockers (ARB) (31.4%), traditional Chinese medicine (23.5%), beta-blockers (21.8%), diuretics (14.9%), nitrates (13.2%), clopidogrel (12%), proton-pump inhibitors (7.2%), angiotensin-converting enzyme inhibitors (ACEI) (6.4%) and calcium dobesilate (5.3%).

Over the 3 years, the regimens of statin (from 41.4% to 44.2%), beta-blockers (from 21.1% to 21.8%), clopidogrel (from 10.4% to 12%) and proton-pump inhibitors (from 6.3% to 7.2%) gradually increased in the elderly. However, the prescriptions of traditional Chinese medicine (from 28% to 23.5%), nitrates (from 13.9% to 13.2%), and ACE inhibitors (from 7.5% to 6.4%) decreased significantly in the elderly (Figure 1).

## 4 Discussion

The direct costs refer to the health expenditures for diabetes regardless of whether these expenditures are borne by the diabetic patients themselves or by private or public payers including the government. The growth of global diabetes health expenditure is significant. The health expenditure of adult diabetic patients (20–79 years old) has increased from US$232 billion in 2007 to US$966 billion in 2021, which means an increase of 316% in 15 years. At the national level in 2021, diabetes-related health expenditures are highest in the United States ($379.5 billion), followed by China and Brazil ($165.3 billion and $42.9 billion, respectively) (IDF DIABETES ATLAS, 2021). The population of elderly diabetic patients is growing fast, posing an obvious burden on population health and economics (Sinclair et al., 2020). In 2017, the average cost of elderly diabetic patients aged over 65 years in the United States was more than $13000, far exceeding the cost of non-elderly diabetic patients (American Diabetes Association, 2018b). About 61% of total healthcare costs for diabetes are incurred by elderly diabetic patients (≥65 years of age).

Based on real-world research, this study analyses the financial costs and medication regimens of older diabetic patients in Beijing, China, from a health insurance perspective. We are devoted to reducing the financial burden and optimizing treatment options for diabetic patients in Beijing in the future. Our previous results showed a gradual decrease in annual medication costs from 2016 to 2018 in all diabetic patients, which could be explained by a more rational medication or the change of medical insurance policy (Guo et al., 2021). In this study, the annual cost for elderly diabetic patients in 2017 was lower than in 2016, the cost in 2018 rebounded again, which may be related to the Zero-Markup Medicine Policy of China implemented in 2017. At the same time, the current situation of comprehensive control of diabetes in elderly patients is worrying, and the complexity of their medication is still on the increasing trend. This result is consistent with the survey in the United States (American Diabetes Association, 2018b).

Older diabetic patients are at higher risk of macrovascular and microvascular complications (Leung et al., 2018). Elderly diabetic patients are more likely to be on multiple medications (Munshi et al., 2012). From this perspective, it is important to select more appropriate antiglycemic drugs to better benefit the patients economically and prohibit the progression of complications. Diabetes management in older patients faces challenges due to extensive variability. Medication analysis in this study found that medication regimens for elderly diabetic patients are becoming complicated, and insulin usage is increasing. Oral combination medication has become the most commonly used treatment option for diabetic patients.

Despite current guidelines recommending a moderate glycemic target in older patients with multiple comorbidities, overtreatment of diabetes in elderly patients remains common (Lipska et al., 2015). The number of medications including antiglycemic drugs increases year by year in diabetic patients in China. However, due to the lack of laboratory data in our study, it is impossible to judge the blood glucose control status of elderly diabetic patients. Insulin could be prescribed for elderly diabetic patients so long as the complexity of the treatment is not overwhelming (Leung et al., 2018). Premixed insulin is the most commonly prescribed insulin in the Chinese diabetic patients. The usage of premixed insulin can reduce the time of injection in elderly diabetic patients while reducing the risk of hypoglycemia in patients with cognitive dysfunction to a certain extent. However, for most elderly diabetic patients, premixed insulin may increase the risk of hypoglycemia (Guo and Xiao, 2024). Long-acting basal insulin analogs are related to a lower frequency of hypoglycemia (Diabetes Canada Clinical Practice Guidelines Expert Committee et al., 2018). Oral agents combined with basal insulin are also well-tolerated in the elderly. This study showed a decreased trend of prescribed premixed insulin with an increased tendency for long-acting and fast-acting insulin.

As the first-line therapy for elderly diabetic patients, metformin is well tolerated if there is a stable renal function (Lipska et al., 2011). However, our results showed that the α-glucosidase inhibitors are still the most commonly prescribed antiglycemic drug in Chinese elderly diabetic patients. Only 48.4% of elderly diabetic patients were using metformin. This could be due to the difference in dietary characteristics between the Chinese and Western populations (Guo et al., 2021). Other reasons for the difference of patients in the prescriptions may be that older people are more likely to have gastrointestinal reactions or with worse renal functions (Schlender et al., 2017). Additional antiglycemic choices should be considered individually according to the guidelines (American Diabetes Association, 2018a).

Medications with a lower risk of hypoglycemia should be chosen preferentially. Sulfonylureas and glinides could increase the risk of hypoglycemia in the elderly. Our 3-year data also show that the treatment plan is being optimized, and the use of sulfonylureas is decreasing year by year in elderly patients. The elderly often suffer from renal insufficiency, so when choosing antiglycemic or antihypertensive drugs, new agents like glucagon-like peptide-1 receptor agonist (GLP-1RA) or sodium-glucose co-transporter-2 inhibitors (SGLT-2i) or medications that can lower urine protein level should be given priority. However, GLP-1RA and SGLT-2i were not included in the Chinese Medical Insurance in 2018.

As far as non-antiglycemic medications are concerned, statin has been the most commonly prescribed drug in the elderly which is consistent with the data on comorbidities. For hypertensive drugs used in elderly diabetic patients, angiotensin-converting-enzyme inhibitors, ARBs, thiazide diuretics, or calcium channel blockers should be considered as initial therapy (Yandrapalli et al., 2018). Elderly diabetic patients with hypertension are always accompanied by cardiovascular diseases (Yandrapalli et al., 2018) and benefit from antihypertensive drugs like beta-blockers. Combinative therapy is always required in the elderly. Our data showed that among the top ten non-antiglycemic drugs, there are five antihypertensive drugs, which further illustrates the degree of complexity in diabetic patients with hypertension. In elderly patients with diabetes combined with hypertension, we often recommend ACEI or ARB drugs as the first choice of antihypertensive agent. At the same time, elderly diabetic patients are often tested positive for urine protein, but the regimens of ACEI and ARB are not satisfactory. In addition, the prescriptions of traditional Chinese medicine are decreasing over the 3 years.

There are some limitations in this study. First, the research information is extracted from all the people registered to participate in medical insurance within the 3 years in Beijing, excluding those who live in Beijing but do not enjoy medical insurance covering diabetes. Besides, our study takes diabetes patients who take medicine continuously as subjects, only insured patients were analyzed, out-of-pocket payers were excluded, which may lead to the exclusion of some patients with diabetes who do not follow the doctor’s advice or lose the follow-up, and leading to the bias of the study population. In addition, this study is retrospective, and there is inevitably potential bias in the data collection process. In our study, we did not take into account indirect medical costs such as hospitalizations and productivity losses, which may have some impact on the results. Second, only 3 years of data are included in this study, the time span is relatively short, which has certain limitations. Considering that comorbidities may overlap, multicollinearity should be tested in future research. Also, new agents like GLP-1RA and SGLT-2i were not available before 2018. No one can deny the fact that GLP-1A and SGLT-2i have been widely used in diabetic patients in Beijing since 2018, so the data from 2016 to 2018, may be somewhat outdated and not fully reflective of the current economic situation and medication use among diabetic patients. In addition, in recent years, new drugs such as Entresto (sacubitril/valsartan) and semaglutide have emerged, potentially influencing treatment patterns and costs. To compare with data of 2016–2018, we are now committed to collect health insurance data in the period of 2019–2021, and analyze the impact of these drugs on elderly patients with diabetes. In the future, long-term database analysis is required to provide new agents for this population.

In conclusion, diabetes management in older patients faces challenges due to extensive variability. Medication analysis in this study found that the current situation of comprehensive control of elderly diabetic patients is worrying, and due to the particularity of the elderly, the complexity of their medication is still on the increasing trend. For the elderly patients with diabetes, especially those with multiple complications, it is important to select more appropriate antiglycemic drugs in order to economically benefit the patients and prohibit the progression of complications. Our research may provide a real world reference basis for drug pricing, insurance policies and the preparation of clinical guidelines for elderly patients with diabetes, which is of certain significance.

## Data Availability

The raw data supporting the conclusions of this article will be made available by the authors, without undue reservation.
