# Supplementary material for: Economic costs and medications for diabetes in older patients in Beijing, China: electronic insurance data analysis
Source: Front Pharmacol. 2025 Apr 7;16:1549244. doi: 10.3389/fphar.2025.1549244 (PMC12009719; doi:10.3389/fphar.2025.1549244)
Supplement: Supplementary file 1 [file Table1.docx]

**Supplementary table 1. Characteristics of included patients**

| **Variable** | | **≥65** | | |
| --- | --- | --- | --- | --- |
|  |  | **2016 (%)** | **2017 (%)** | **2018 (%)** |
| **Gender (%)** | Male | 118,388 (49.7) | 130,718 (50.1) | 143,387 (50.2) |
|  | Female | 119,652 (50.3) | 130,382 (49.9) | 141,991 (49.8) |
| **Hypertension (%)** | Absent | 28,334 (11.9) | 31,669 (12.1) | 35,279 (12.4) |
|  | Present | 209,706 (88.1) | 229,431 (87.9) | 250,099 (87.6) |
| **Coronary heart**  **disease (%)** | Absent | 41,753 (17.5) | 46,387 (17.8) | 51,460 (18.0) |
|  | Present | 196,287 (82.5) | 214,713 (82.2) | 233,918 (82.0) |
| **Dyslipidemia (%)** | Absent | 62,058 (26.1) | 67,734 (25.9) | 73,774 (25.9) |
|  | Present | 175,982 (73.9) | 193,366 (74.1) | 211,604 (74.1) |
| **Stroke (%)** | Absent | 119,136 (50.0) | 131,740 (50.5) | 145,280 (50.9) |
|  | Present | 118,904 (50.0) | 129,360 (49.5) | 140,098 (49.1) |
| **Chronic lung**  **disease (%)** | Absent | 134,457 (56.5) | 147,704 (56.6) | 161,698 (56.7) |
|  | Present | 103,583 (43.5) | 113,396 (43.4) | 123,680 (43.3) |
| **Osteoporosis (%)** | Absent | 158,775 (66.7) | 174,387 (66.8) | 191,140 (67.0) |
|  | Present | 79,265 (33.3) | 86,713 (33.2) | 94,238 (33.0) |
| **Diabetic peripheral neuropathy (%)** | Absent | 161,584 (67.9) | 177,983 (68.2) | 195,353 (68.5) |
|  | Present | 76,456 (32.1) | 83,117 (31.8) | 90,025 (31.5) |
| **Diabetic nephropathy (%)** | Absent | 210,772 (88.5) | 231,323 (88.6) | 253,011 (88.7) |
|  | Present | 27,268 (11.5) | 29,777 (11.4) | 32,367 (11.3) |
| **Diabetic retinopathy (%)** | Absent | 209,588 (88.0) | 229,808 (88.0) | 251,271 (88.0) |
|  | Present | 28,452 (12.0) | 31,292 (12.0) | 34,107 (12.0) |
