# Supplementary material for: Economic costs and medications for diabetes in older patients in Beijing, China: electronic insurance data analysis
Source: Front Pharmacol. 2025 Apr 7;16:1549244. doi: 10.3389/fphar.2025.1549244 (PMC12009719; doi:10.3389/fphar.2025.1549244)
Supplement: Supplementary file 2 [file Table2.docx]

**Supplementary Table 2. Insulin Use Over 3-Year Period**

| **Type of Insulin** | **Uesd** | **≥65** | | | | |
| --- | --- | --- | --- | --- | --- | --- |
|  |  | **2016 (%)** | **2017 (%)** | **2018 (%)** | **χ2** | **p value** |
| Fast-acting | No | 92.3 | 90.4 | 88.7 | 603.7177 | <.0001 |
|  | Yes | 7.7 | 9.6 | 11.3 |  |  |
| Short-acting | No | 85.5 | 86.8 | 87.7 | 163.5633 | <.0001 |
|  | Yes | 14.5 | 13.2 | 12.3 |  |  |
| Intermediate-acting | No | 84.0 | 85.5 | 86.8 | 265.6264 | <.0001 |
|  | Yes | 16.0 | 14.5 | 13.2 |  |  |
| Long-acting | No | 79.8 | 76.6 | 73.5 | 893.2233 | <.0001 |
|  | Yes | 20.2 | 23.4 | 26.5 |  |  |
| Premixed | No | 40.0 | 41.3 | 43.8 | 267.6268 | <.0001 |
|  | Yes | 60.0 | 58.7 | 56.2 |  |  |
